# DGEclust: differential expression analysis of clustered count data

**DOI:** 10.1186/s13059-015-0604-6

**Published:** 2015-02-20

**Authors:** Dimitrios V Vavoulis, Margherita Francescatto, Peter Heutink, Julian Gough

**Affiliations:** Department of Computer Science, University of Bristol, Bristol, UK; Genome Biology of Neurodegenerative Diseases, Deutsches Zentrum für Neurodegenerative Erkrankungen, Tübingen, Germany

## Abstract

We present a statistical methodology, *DGEclust*, for differential expression analysis of digital expression data. Our method treats differential expression as a form of clustering, thus unifying these two concepts. Furthermore, it simultaneously addresses the problem of how many clusters are supported by the data and uncertainty in parameter estimation. *DGEclust* successfully identifies differentially expressed genes under a number of different scenarios, maintaining a low error rate and an excellent control of its false discovery rate with reasonable computational requirements. It is formulated to perform particularly well on low-replicated data and be applicable to multi-group data. *DGEclust* is available at http://dvav.github.io/dgeclust/.

**Electronic supplementary material:**

The online version of this article (doi:10.1186/s13059-015-0604-6) contains supplementary material, which is available to authorized users.

## Background

Next-generation sequencing (NGS) and high-throughput sequencing are revolutionary tools for the study of the genome, epigenome and transcriptome in a multitude of organisms (including humans) by allowing the relatively rapid production of millions of short sequence tags, which mirror particular aspects of the molecular state of the biological system of interest. A common application of NGS is the study of the transcriptome, which involves a family of methodologies, such as RNA sequencing (RNA-seq) [1], cap analysis of gene expression (CAGE) [2], serial analysis of gene expression (SAGE) [3] and others. Most published studies on the statistical analysis of count data generated by NGS have focused on the themes of experimental design [4], normalisation [5,6] and the development of tests for differential expression [7-9]. Surprisingly, not much attention has been paid to cluster analysis.

Clustering is considered an important tool in the study of genomic data and it has been used extensively in the analysis of microarrays [10-12] (see [13] for a review of different clustering methods). It involves grouping together the expression profiles of different genes across different points in time, treatments and tissues, such that expression profiles in the same group are more similar in some way to each other than to members of other groups. Genes that are clustered together across samples exhibit co-related expression patterns, which might indicate co-regulation and involvement of these genes in the same cellular processes [14]. Moreover, whole samples of gene expression profiles can be clustered together, indicating a particular macroscopic phenotype, such as cancer [15].

A large class of clustering methods relies on the definition of a distance metric, which quantifies the similarity between any two gene expression data points. Subsequently, clusters are formed, such that the distance between any two data points in the same cluster is minimised. Typical methods in this category are *k*-means clustering and self-organising maps [13]. Another important category includes model-based clustering algorithms. In this case, the whole gene expression dataset is modelled as a random sample from a finite mixture of probability distributions, where each component of the mixture corresponds to a distinct cluster. The parameters of each component in the mixture (e.g. mean and variance) are usually estimated using an expectation-maximisation algorithm [13]. Hierarchical clustering is yet a third type of clustering methodology, which is particularly suited for modelling genomic (often hierarchically organised) data. It generates a hierarchical series of nested clusters, which can be represented graphically as a *dendrogram*. This stands in contrast to partition-based methods (e.g. *k*-means or self-organising maps), which decompose the data directly into a finite number of non-overlapping clusters [13].

In this article, we present a model-based statistical methodology and associated software (*DGEclust*) for clustering digital expression data and we show how it can be used in differential expression analysis. From a theoretical standpoint, the significance of the proposed methodology stems from its unification of differential expression and clustering (i.e. it treats differential expression as a particular clustering configuration of the data). This makes it possible to bypass the need for a particular statistical test when inferring differential expression or for multiple hypothesis testing correction, such as the Benjamini–Hochberg procedure. From a statistical point of view, the proposed methodology is important because it simultaneously addresses the problem of model selection (i.e. how many clusters are supported by the data) and uncertainty (i.e. the error associated with estimating the number of clusters and the parameters of each cluster). This is made possible by exploiting a hierarchical Dirichlet process mixture model (HDPMM) [16,17], a statistical framework, which has been applied in the past for clustering microarray data [18,19], for multi-population haplotype inference [20], for integrating heterogeneous genomic datasets [21,22] and for modelling multiple text corpora [23]. In our version of the HDPMM, individual expression profiles are drawn from the negative binomial distribution (as, for example, in [24-27]) and parameter estimation is achieved using a novel, fast blocked Gibbs sampler, which efficiently processes large datasets (e.g. with more than 20K genes). We show how the output of our clustering algorithm can be used in differential expression analysis and, using simulated data and actual experimental data (RNA-seq and CAGE) from a range of species, we demonstrate improved performance, compared to popular alternative methods. An early version of the proposed methodology has been presented previously in poster format and in [28].

## Results and discussion

### Description of the model

Formally, the production of count data using NGS assays can be thought of as random sampling of an underlying population of cDNA fragments. Thus, the counts for each tag describing a class of such fragments can, in principle, be modelled using the Poisson distribution, whose variance is, by definition, equal to its mean. However, it has been shown that, in real count data of gene expression, the variance can be larger than what is predicted by the Poisson distribution [29-32]. An approach that accounts for the so-called over-dispersion in the data is to adopt quasi-likelihood methods, which augment the variance of the Poisson distribution with a scaling factor, thus dropping the assumption of equality between the mean and variance [33-36]. An alternative approach is to use the negative binomial distribution, which is derived from the Poisson, assuming a gamma-distributed rate parameter. The negative binomial distribution incorporates both a mean and a variance parameter, thus modelling over-dispersion in a natural way [24-26]. For this reason, in this article we use the negative binomial distribution for modelling count data.

We indicate the number of reads for the *i*th feature (e.g. gene) at the *j*th sample/library with the variable *y*_*ij*_. Each library *j* is assigned to a group of libraries *l*=*λ*(*j*), based on prior information on tissue, experimental condition, disease state, etc. There are a total of *N* genes, *M* libraries and *L* groups of libraries, where 2≤*L*≤*M*. We assume that *y*_*ij*_ is distributed according to a negative binomial distribution, with mean *m*_*ij*_ and dispersion parameter *ϕ*_*i*_:

(1) $$ y_{ij}|\phi_{i}, \mu_{i}, \beta_{i\lambda(j)} \sim \text{NegBin}\left(m_{ij}, \phi_{i}\right)   $$

where log(*m*_*ij*_)= log(*c*_*j*_)+ log(*μ*_*i*_)+*β*_*iλ*(*j*)_. In the previous expression, *c*_*j*_ is a known *a priori* normalising factor for library *j*, *μ*_*i*_ is the mean expression level for gene *i* and *β*_*iλ*(*j*)_ is the fold-change of the mean expression level of gene *i* in group *λ*(*j*). A fold-change equal to 0 indicates no change in the mean expression level for feature *i* in group *λ*(*j*), while a value larger (smaller) than 0 indicates over(under)-expression.

The variance for expression profile *y*_*ij*_, $\sigma ^{2}_{\textit {ij}}=m_{\textit {ij}}+\phi _{i} m_{\textit {ij}}^{2}$, is always larger than the mean by the quantity $\phi _{i} m_{\textit {ij}}^{2}$. Thus, the negative binomial distribution can be thought of as a generalisation of the Poisson distribution, which accounts for over-dispersion.

It follows that to specify the above model fully, we need to know the parameters *ϕ*_*i*_, *μ*_*i*_ and *β*_*iλ*(*j*)_ for each gene *i* and group of samples *λ*(*j*). We also need to compute the normalising factors *c*_*j*_ from the data (see [37] for a review of available normalisation methods).

#### Information sharing between genes

A common limitation in experiments using NGS technologies is the low number or even absence of biological replicates, which complicates the statistical analysis of digital expression data. One way to compensate for small sample sizes is to assume that all genes share the same variance [30]. A less restrictive approach is to implement some type of information sharing between genes, which permits the improved estimation of gene-specific parameters (e.g. the dispersion parameters) by pooling together genes with similar expression profiles [24-26]. In this article, information sharing between genes and between samples is introduced in a natural way through the use of priors for the parameters of the negative binomial distribution. In this and the following section, we use Dirichlet process priors for modelling the fold-changes *β*_*iλ*(*j*)_, followed by giving the priors for the *ϕ*_*i*_ and *μ*_*i*_ parameters.

Within each sample group *l*=*λ*(*j*), we assume that the gene-specific fold-changes {*β*_*il*_} are random and distributed according to a prior distribution *G*_*l*_, i.e.

(2) $$ \beta_{il}|G_{l} \sim G_{l}   $$

Furthermore, we assume that *G*_*l*_ is itself randomly sampled from a *Dirichlet process* with positive *concentration parameterγ*_*l*_ and *base probability* distribution *G*_0_ [16]:

(3) $$ G_{l}|\gamma_{l},G_{0} \sim \text{DP}(\gamma_{l},G_{0})   $$

**Figure 1:**
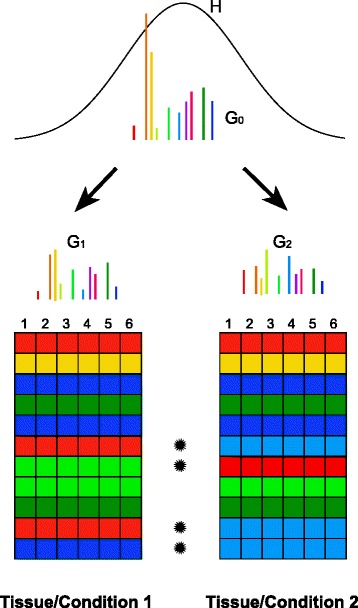
**Information sharing between genes and between sample classes.** The statistical model in *DGEclust* internally models the counts for each gene *i* in each library *j* as random variables sampled from a negative binomial distribution with gene-specific parameters *μ*_*i*_ and *ϕ*_*i*_ and gene- and experimental condition- (or tissue-) specific log-fold-changes *β*_*il*_. Different genes within the same condition *l* may share the same log-fold-changes, which are randomly sampled from discrete, condition-specific random distributions (*G*_1_ and *G*_2_ in the figure). This imposes a clustering effect on genes in each experimental condition; genes in the same cluster have the same colour in the figure, while the probability of each cluster is proportional to the length of the vertical lines in distributions *G*_1_ and *G*_2_. The discreteness of *G*_1_ and *G*_2_ is because they are random samples themselves from a Dirichlet process with global base distribution *G*_0_, which is also discrete. Since *G*_0_ is shared among all experimental conditions, the clustering effect extends between them, i.e. a particular cluster may include genes from the same and/or different experimental conditions. Finally, *G*_0_ is discrete, because it too is sampled from a Dirichlet process with base distribution *H*, like *G*_1_ and *G*_2_. If the expression profiles of a particular gene belong to two different clusters across two experimental conditions, then this gene is considered differentially expressed (see rows marked with stars in the figure).

Dirichlet process priors are distributions over distributions and they have become a popular choice in Bayesian inference studies, since they provide an elegant solution to the problem of determining the correct number of components in mixture models. Standard theoretical results [38] state that a sample *G*_*l*_ from Equation 3 is a discrete distribution with probability one over a countably infinite set of *β*s. Large values of *γ*_*l*_ lead to a large number of similarly likely values of *β*, while small values of this parameter imply a small number of highly probable values of *β*. This and Equation 2 imply that the fold-changes *β*_*il*_ within the *l*th group of samples are not all distinct. Different genes may share the same value of *β* or, in other words, genes are grouped in a (not known in advance) number of clusters, based on the value of *β* they share. Equivalently, the expression profiles of different groups of genes are drawn from different negative binomial distributions, each characterised by its own unique value of *β*. This clustering effect is illustarted in Figure 1.

#### Information sharing between samples

Up to this point, we have considered clustering of genes within the same group of samples, but not across groups of samples (e.g. tissues or conditions). However, in a given dataset, each cluster might include gene expression profiles from the same, as well as from different groups of samples. In other words, clusters are likely shared between samples that belong to different groups. This sharing of information between sample groups can be expressed naturally in the context of HDPMMs [16]. Following directly from the previous section, we assume that the base distribution *G*_0_ is itself random and sampled from a Dirichlet process with a global scaling parameter *δ* and a global base distribution *H*:

(4) $$ G_{0}|\delta,H \sim \text{DP}(\delta,H)   $$

The above expression implies that *G*_0_ is (like each *G*_*l*_) discrete over a countably infinite set of atoms *βk*′, which are sampled from *H*, i.e. *βk*′∼*H*. Since *G*_0_ is the common base distribution of all *G*_*j*_, the atoms *βk*′ are shared among all samples, yielding the desired information sharing across samples (see Figure 1).

#### Generative model

In summary, we have the following hierarchical model for the generation of a matrix of digital gene expression data (see also Figure 1):

(5) $$\begin{array}{@{}rcl@{}} H & \equiv & \text{Normal}\left(\mu_{\beta},\sigma^{2}_{\beta}\right) \\ G_{0}|\delta,H & \sim & \text{DP}(\delta,H)  \\ G_{l}|\gamma_{l},G_{0} & \sim & \text{DP}(\gamma_{l},G_{0}) \\ \beta_{il} & \sim & G_{l} \\ \log(\phi_{i}) & \sim & \text{Normal}\left(\mu_{\phi},\sigma^{2}_{\phi}\right) \\ y_{ij}|\phi_{i}, \mu_{i}, \beta_{il} & \sim & \text{NegBin}\left(c_{j}\mu_{i}e^{\beta_{il}}, \phi_{i}\right)  \end{array} $$

where *l*≡*λ*(*j*). Notice that the base distribution *H*, which provides the global prior for sampling the atoms $\beta ^{\prime }_{k}$, was assumed normal with mean *μ*_*β*_ and variance $\sigma ^{2}_{\beta }$. Similarly, the logarithm of the dispersion parameters *ϕ*_*i*_ was assumed normal with mean *μ*_*ϕ*_ and variance $\sigma ^{2}_{\phi }$ (see, for example, [39] for a justification of this choice). The mean expression levels *μ*_*i*_ are modelled as $\mu _{i} = (1 - p_{i})p_{i}^{-1}\phi _{i}^{-1}$, where *ϕ*_*i*_ is sampled as above and *p*_*i*_∼Beta(0.5,0.5). This formulation greatly facilitates the posterior inference of *μ*_*i*_ given *ϕ*_*i*_, since it involves sampling directly from well-known distributions (see Additional File 1 for more details).

### Inference

The definition of the HDPMM in Equations 5 is implicit. To facilitate posterior inference, an equivalent constructive representation of the above model has been introduced in [23], using Sethuraman’s stick-breaking representation of a draw from a Dirichlet process [38]. This representation introduces a matrix of indicator variables *z*={*z*_*il*_}, where each element of the matrix, *z*_*il*_, indicates which cluster the *i*th log-fold-change in the *l*th group of samples (i.e. *β*_*il*_) belongs to. Two different *β*’s, *β*_*il*_ and $\beta _{i^{\prime }l^{\prime }}$, belong to the same cluster if and only if their indicator variables, e.g. *z*_*il*_ and $z_{i^{\prime }l^{\prime }}$, are equal. A major aim of Bayesian inference in the above model, is to calculate the posterior distribution p(*z*|*y*) of matrix *z* given the data matrix *y*.

One approach to estimate this distribution is by using Markov chain Monte Carlo (MCMC) methods, which generate a chain of random samples as a numerical approximation to the desired distribution. We have developed a blocked Gibbs sampler in the software package *DGEclust*, which efficiently generates new samples from the posterior p(*z*|*y*). The algorithm is an extension of the method presented in [28,40] for inference in non-HDPMMs and its advantage is that it samples each element of *z* independently of all others. This not only results in very fast convergence, but it also allows the algorithm to be implemented in vectorised form, which takes advantage of the parallel architecture of modern multicore processors and potentially permits application of the algorithm on very large datasets. Alternative MCMC methods, which are developed on the basis of the popular Chinese restaurant franchise representation of the Hierarchical Dirichlet Process (HDP) [16,41], do not enjoy the same advantage since they are restricted because sampling each indicator variable is conditioned on the remaining ones, thus all of them must be updated in a serial fashion. Details of the algorithm are given as supplementary material (see Additional file 1).

### Testing for differential expression

Assuming that the above algorithm has been applied on a digital expression dataset *y* and a sufficiently large chain of samples $\phantom {\dot {i}\!}z^{(T_{0}+1)},z^{(T_{0}+2)},\ldots,z^{(T_{0}+T)}$ – which approximates the posterior p(*z*|*y*) – has been generated, we show how these samples can be used for differential expression analysis. We consider two classes of samples, A and B, which might represent, for example, two different tissues or experimental conditions.

A particular gene is said to be *not* differentially expressed (DE), if its expression measurements in classes *A* and *B* belong to the same cluster. In more formal language, we state that the posterior probability *π*_*i*_ that gene *i* is *not* DE given data *y* is equal to the conditional probability p(*z*_*iA*_=*z*_*iB*_|*y*) that the indicator variables of feature *i* in sample classes *A* and *B* have the same value. This probability can be approximated as a simple average over the previously generated MCMC samples $\left \{z^{T_{0}+t}\right \}_{t=1}^{T}$:

(6) $$ \pi_{i} = \frac{\sum_{t=T_{0}+1}^{T_{0}+T} 1\!\!1\left(z_{iA}^{(t)}=z_{iB}^{(t)}\right)}{T}   $$

where 1 1(·) is equal to 1 if the expression inside the parentheses is true and 0 otherwise. Given a threshold $\tilde \pi $, we can generate a set  of potentially DE features with probabilities less than this threshold, i.e. $\mathcal {D}=\{i: \pi _{i}\le \tilde \pi \}$, where *π*_*i*_ is calculated as in Equation 6 for all *i*.

As observed in [42], the quantity *π*_*i*_ measures the conditional probability that including the *i*th gene in list  is a Type I error, i.e. a false discovery (FD). This useful property makes possible the calculation of the conditional false discovery rate (FDR) as follows:

(7) $$ \text{FDR}(\tilde\pi) = \frac{\sum_{i} \pi_{i} 1\!\!1\left(\pi_{i}\le\tilde\pi\right)} {\sum_{i} 1\!\!1\left(\pi_{i}\le\tilde\pi\right)}   $$

From Equation 7, it can be seen that  always has an FDR at most equal to $\tilde \pi $. Alternatively, one can first set a target FDR, say tFDR, and then find the maximum possible value of $\tilde \pi $, such that $\text {FDR}(\tilde \pi) \le \text {tFDR}$.

Notice that, unlike alternative approaches, which make use of gene-specific *P* values, this methodology does not require any correction for multiple hypothesis testing, such as the Benjamini–Hochberg procedure. Although the computation of FDR using Equation 7 is approximate (since it depends on the accuracy of the calculation of *π*_*i*_ using Equation 6), it is reasonable to assume that the error associated with this approximation is minimised, if sufficient care is taken when post-processing the MCMC samples generated by the Gibbs sampler.

### Application to simulated data

To assess the performance of our methodology, we applied it on simulated and actual experimental count data and we compared our results against those obtained from popular software packages, namely *DESeq*/*DESeq2*, *edgeR* and *baySeq*.

First, we applied our algorithm on simulated data, which provides the advantage that we can control the exact conditions under which the data was generated, including the true differential expression state of each gene. For this purpose, we used the function *generateSyntheticData* from the independent *Bioconductor* package *compcodeR* [43]. Data generated using this approach follow the negative binomial distribution, with mean and dispersion parameters estimated from actual experimental data [44,45]. Each simulated dataset included 10K genes under two different experimental conditions, with the proportion of DE genes set to either 0%, 10% or 30% (0, 1,000 or 3,000 DE genes, respectively). In addition, we considered simulated data where 50% of the genes were generated from the Poisson distribution (and, therefore, were not over-dispersed), as well as simulated data which included outliers, i.e. genes with unusually high or low counts. The outliers were introduced by multiplying with probability 5% each observed count independently with a randomly generated factor between 5 and 10. Finally, we evaluated the effect of varying the per-condition sample size from 2 to 4 and 8, since small sample sizes reflect the design of most current sequencing experiments. Under each unique simulation setting, we independently generated three different datasets; each evaluated method was applied separately on each dataset and the aggregated results for each simulation setting were reported. All parameters of *generateSyntheticData* were left as their default values, unless stated otherwise.

Following the prototypical study in [9], we compared the performance of *DGEclust* and the other aforementioned methods under the experimental conditions encapsulated by the synthetic data using the following criteria: (a) first, we compared the ability of all methods to identify DE genes using as performance measure the area under the receiver operating characteristic (ROC) curve (AUC) for each method, (b) second, we compared the ability of all methods to keep a low number of Type I errors using FD curves as performance measures and also by measuring the number of Type I errors each method returned given a fixed significance threshold, (c) third, we assessed the ability of all methods to keep a low FDR given a relatively large pre-specified FDR threshold. Furthermore, we applied all methods on actual RNA-seq data from a number of species, as well as on a large CAGE dataset obtained from five different regions of human brains. Details for each category of experiment are given in the following sections.

#### *DGEclust* successfully identifies differentially expressed genes under a number of different scenarios

We first evaluated the comparative ability of *DGEclust* to identify truly DE genes. All examined methods rank each gene by providing *P* values (*edgeR* and *DESeq*/*DESeq2*) or posterior probabilities (*DGEclust* and *baySeq*). Given a threshold score, genes on opposite sides of the threshold are tagged as DE or non-DE, accordingly. In an artificial dataset, the genes that were simulated to be DE are considered to be the true positive group, while the remaining genes are considered the true negative group. By computing the false positive rate (FPR) and the true positive rate (TPR) for all possible score thresholds, we can construct ROC and FD curves for each examined method. The AUC is a measure of the overall discriminative ability of a method (i.e. its ability to classify correctly features as DE or non-DE).

**Figure 2:**
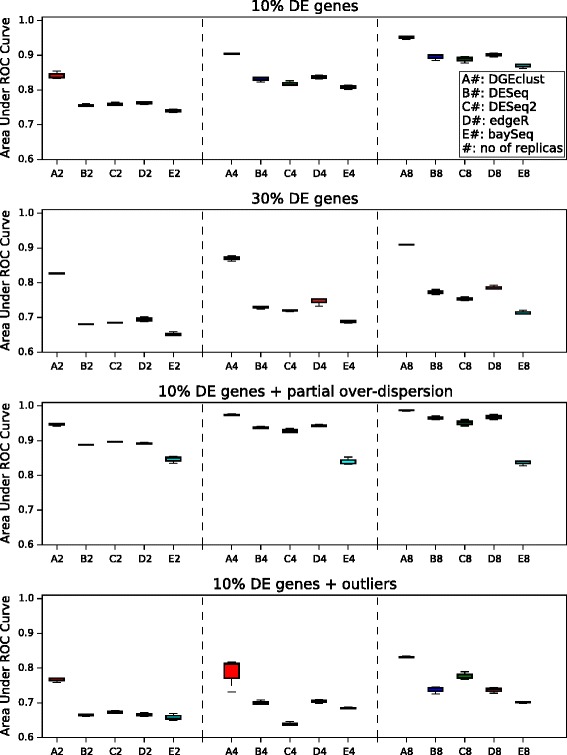
**Comparison of different methods.** The area under the receiver operating characteristic curve is used as the performance measure. The box plots summarise the results obtained across three independent synthetic datasets for four different simulation settings. Each dataset included 10K genes and results across 2, 4 and 8 biological replicates are reported. *DGEclust* shows improved performance in comparison to other methods in all of the examined cases, particularly in the presence of a large proportion of differentially expressed genes (30%) and small sample sizes (*n*=2). The inclusion of non-over-dispersed genes significantly improves the performance of all methods, but the inclusion of outliers has the opposite effect. Still, *DGEclust* remains top-ranked among the alternative methods with respect to AUC scores. AUC, area under the receiver operating characteristic curve; DE, differentially expressed; ROC, receiver operating characteristic.

Our results are summarised in Figure 2. As expected, the performance of all methods improves as the sample size in each dataset increases from 2 to 4 to 8. When only over-dispersed genes are present and only 10% of them are DE (top panel), *DGEclust* clearly demonstrates the best performance with an AUC score larger than 80%, followed by *DESeq*/*DESeq2*, *edgeR* and, finally, *baySeq*. The same trend is observed when we examine datasets with 30% DE genes (second panel), but this time the difference between *DGEclust* and the other methods is even more prominent. This is because the performance of *edgeR*, *DESeq*/*DESeq2* and *baySeq* is negatively affected by the increased proportion of DE genes, while the performance of *DGEclust* remains essentially the same.

The negative effect of DE gene composition on *DESeq*, *edgeR* and *baySeq*, when datasets with large proportions of DE genes were considered, has already been observed [9] and attributed to an increased proportion of false positives due to the failure of normalisation to capture the effect of the possibly asymmetric distribution of DE genes fully. Here, we provide an alternative explanation that this effect might be an intrinsic characteristic of each classification method, since *DGEclust* does not seem to be affected by the presence of this asymmetry in the data, although it adopts the same normalisation method as *DESeq*.

We further evaluated the effect of introducing non-over-dispersed genes or outliers, as outlined above. When the fraction of Poisson distributed genes was increased from 0 to 50% (third panel), we observed a significant increase in the AUC for all methods. *DGEclust* ranked first in all cases, but *DESeq*/*DESeq2* and *edgeR* followed closely, particularly at large sample sizes (*n*=8). The introduction of outliers with abnormally high or low counts (bottom panel), reduced the AUC for all methods, but again *DGEclust* ranked first among the alternatives for all sample sizes.

#### *DGEclust* maintains a low rate of Type I errors

**Figure 3:**
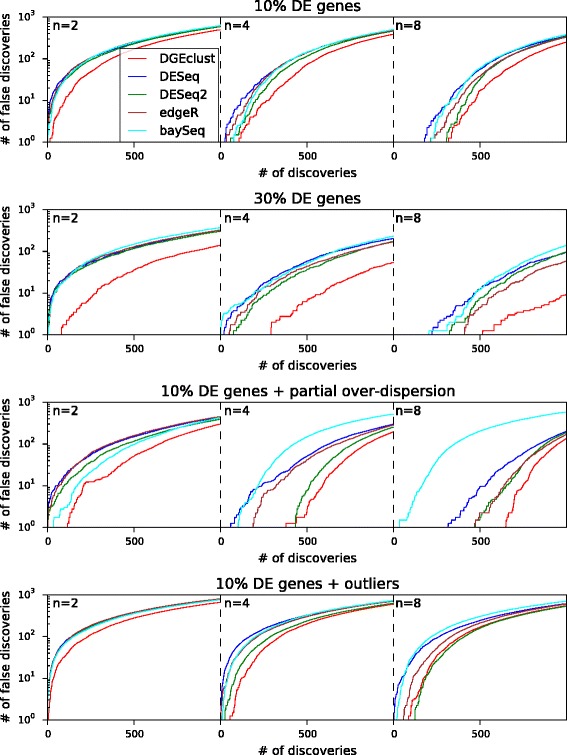
**False discovery curves for all methods across the first 1,000 discoveries.** The illustrated false discovery curves are averages over three independent repetitions of each synthetic dataset. *DGEclust* clearly keeps a lower number of false discoveries in comparison to the other methods in all cases. There is a single exception in the presence of outliers and at large sample sizes (*n*=8), where *DESeq2* appears to be marginally better than *DGEclust* over the first 500 discoveries. DE, differentially expressed.

Next, we evaluated the ability of *DGEclust* and the other methods to control the rate of Type I errors. First, we assessed this ability through the construction of FD curves, which illustrate the number of false positives as a function of the total number of positives (i.e. as the decision threshold increases). Mean FD curves for each of the cases examined in Figure 2 are illustrated in Figure 3. We measure the false positives among the first 1,000 top-ranked discoveries. Observe that when only over-dispersed data with either small (top panel) or large (second panel) numbers of DE genes are considered, *DGEclust* always keeps the number of false positives among the top discoveries below that of the other evaluated methods. As with the AUC scores, this is more prominent when a large number of DE genes (30%) is considered and it holds at all sample sizes. When examining datasets including non-over-dispersed genes (third panel), *DGEclust* still performs better than alternative methods, at all sample sizes. For datasets with outliers (bottom panel), the difference between *DGEclust* and the other methods becomes less prominent and effectively disappears at large sample sizes (*n*=8), at which point *DESeq2* also demonstrates good performance.

**Figure 4:**
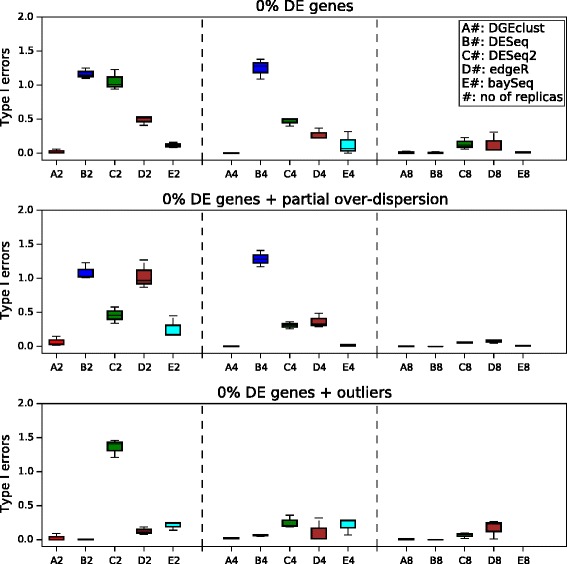
**Type I errors for all methods at a pre-specified significance threshold.** The box plots summarise results across three independently obtained simulated datasets for three different simulation settings. In all cases, exactly zero genes were truly differentially expressed. To make possible a comparison between methods that return *P* values (*DESeq*/*DESeq2* and *edgeR*) and those that return posterior probabilities (*DGEclust* and *baySeq*), we report the Type I error rate at a relatively high false discovery rate, FDR = 10%. In all cases, *DGEclust* maintains a minimal Type I error rate, particularly for small sample sizes (*n*=2). DE, differentially expressed.

In a second stage, we examined the ability of all methods to control the number of Type I errors at a pre-specified threshold level in the absence of any truly DE genes (see Figure 4). To make possible a comparison between methods that return *P* values (*DESeq*/*DESeq2* and *edgeR*) and methods that return posterior probabilities (*DGEclust* and *baySeq*) and since all methods provide an estimation of the FDR, we compared the number of Type I errors (as a proportion of the total number of genes) each method made at a relatively high pre-specified FDR = 0.1. In all datasets not including outliers (top two panels), *DGEclust* had the lowest proportion of Type I errors at small sample sizes (*n*=2 and *n*=4), followed closely by *baySeq*. At large sample sizes (*n*=8), *DGEclust*, *DESeq* and *baySeq* performed similarly, followed closely by *DESeq2* and *edgeR*. After the inclusion of outliers (bottom panel), *DGEclust* again had the lowest Type I error rate along with *DEseq* at all sample sizes.

#### *DGEclust* retains excellent control of its false discovery rate

Furthermore, we evaluated the ability of *DGEclust* and the other methods to control the FDR at a pre-specified level. For all methods, we calculated the true FDR as the fraction of discoveries that were false at a pre-specified significance threshold equal to 0.1. We labelled as *discoveries* all features satisfying the condition that the adjusted *P* values (*DESeq*/*DESeq2*) or estimated FDRs (*DGEclust*, *baySeq* and *edgeR*) were less than or equal to the aforementioned significance level. If no discoveries were identified, the corresponding true FDR was assumed undefined.

**Figure 5:**
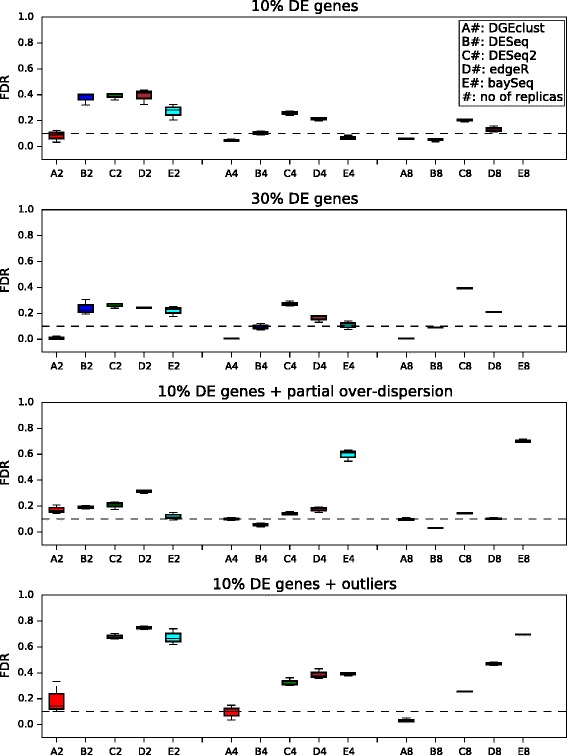
**False discovery rates for all methods at a pre-specified significance threshold.** The box plots summarise the FDRs obtained across three independently obtained simulated datasets at four different simulation settings at an imposed significance level of 10%. The ability of all methods to control their FDR increases with the sample size. In all cases, *DGEclust* demonstrates excellent control over its FD, particularly at small sample sizes (*n*=2). Interestingly, in the presence of outliers, *DGEclust* is the only method that keeps its FDR at or below the pre-specified threshold at all sample sizes. DE, differentially expressed; FD, false discovery; FDR, false discovery rate.

Our results are summarised in Figure 5. In all cases, *DGEclust* was the top or among the top-performing methods in terms of controlling the FDR at a pre-specified level. As a general observation, increasing the number of replicates per condition from 2 to 4 to 8, clearly increased the ability of all methods to control the FDR. *DGEclust* demonstrated excellent control of its FDR, keeping it close to or below the pre-specified significance threshold, in all examined cases. When no outliers and only over-dispersed genes were considered (top two panels), *DGEclust* was the only method that kept the FDR below the pre-specified threshold at low sample sizes (*n*=2). At larger sample sizes (*n*=4,8), *DESeq* appears to reduce this gap and in one case (top panel, *n*=8), it performs as well as *DGEclust*. In the presence of non-over-dispersed genes (third panel), *DGEclust* still retains its FDR at (*n*=4,8) or slightly above (*n*=2) the pre-specified threshold, although it is now marginally overtaken by *baySeq* (*n*=2) or *DESeq* (*n*=4,8). When genes with unusually low or high counts were considered (bottom panel), *DGEclust* managed to retain excellent control of its FDR, even at small sample sizes, and with quite significant differences from all alternative methods. *DESeq* did not call any DE genes in this case and its FDR was deemed undefined.

### *DGEclust* demonstrates top performance for low-replicated data from a range of species

In addition to simulated data, we also tested our method on RNA-seq data from mouse, rat, worm and fly and on CAGE data from humans. The RNA-seq data were obtained from the online resource *ReCount* [46] and they are briefly described below.

The mouse data [47] consisted of two groups of libraries, controls versus CUG-BP1 knockdown myoblasts. Each group included two biological replicates (*n*=2) and, after filtering out all rows with zero count sum, a total of 9,502 genes. Similarly, the rat data [48] consisted of two groups (controls versus subjects with chronic neuropathic pain induced by spinal nerve ligation of the neighbouring L5 spinal nerve) with two biological replicates per group (*n*=2) and, after filtering as above, 17,125 genes. The filtered (as above) worm data [49] consisted of two groups (L1LIN35-1cap1 versus L4MALE6cap2) with two replicates each (*n*=2) and 19,430 genes. Finally, the fly data [50] consisted of two groups, adult males and females, with each group containing three individuals, 1, 5 and 30 days old (*n*=3). After removing all rows with zero count sum across all samples, the filtered dataset included 13,188 genes. For more details about how these data were obtained, the reader is referred to [46] and the original publications.

The CAGE dataset was prepared according to the standard Illumina protocol described in [51] and it consisted of 25 libraries isolated from five brain regions (caudate nucleus, frontal lobe, hippocampus, putamen and temporal lobe) from five human donors (*n*=5) and it included 23,448 features, i.e. tag clusters representing promoter regions (see Materials and methods for more details). In this section, we examine only the libraries from the caudate nucleus and frontal lobe, but we include all libraries in our analysis in the next section.

As in [52], we used the log-fold-change between different groups of samples to establish a ground truth on which we based our subsequent method comparisons. Specifically, a gene was considered DE if the absolute log _2_ fold-change ratio of its normalised mean expression across all replicas between two conditions exceeded a threshold value of 2 (corresponding to a 4× change in expression) and non-DE if this ratio was smaller than 1/2 (corresponding to changes in expression less than approximately 41*%*). All intermediate cases were considered undefined and they were ignored during computation of ROC curves. For CAGE data, we further validated this approach of approximating the true state of differential expression for each gene by adopting the biological homogeneity index (BHI) [53] as an additional proxy of the unknown ground truth (see details in the next section).

**Figure 6:**
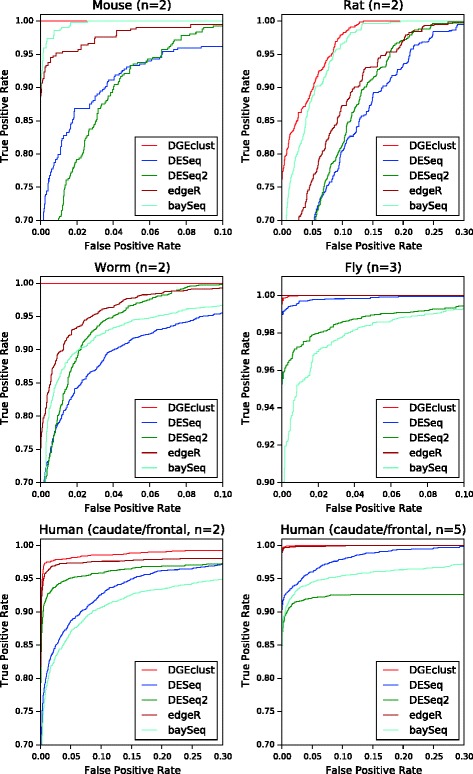
**Comparison of ROC curves from different methods.** The methods are applied to RNA-seq or CAGE data from a number of different species. In all cases, a ground truth was established by considering the absolute value of the log2 ratio of the mean expression across all replicas between two conditions [52]. *DGEclust* demonstrates excellent performance in all cases. At small sample sizes (*n*=2), it is ranked at the top, while for larger sizes (*n*=3 or *n*=5), it performs similarly to *edgeR*. CAGE, cap analysis of gene expression; RNA-seq, RNA sequencing; ROC, receiver operating characteristic.

The performance of all tested methods was excellent, reaching a TPR larger than 70% at minimal (≪0.01) FPRs (Figure 6). The two top-performing methods were *DGEclust* and *edgeR* (worm, fly and human) or *baySeq* (mouse and rat). In several cases (mouse, worm, fly and human with *n*=5), *DGEclust* achieved almost a perfect classification and in all cases it was the only method that consistently demonstrated top performance. It is interesting to observe that while *DGEclust* is clearly the best method at small sample sizes (*n*=2), at larger sample sizes (fly with *n*=3 and human with *n*=5) *edgeR* also performed equally well. Based on this evidence, we conclude that *DGEclust* has a clear advantage compared to alternative methods at low replication levels.

### *DGEclust* is applicable to multi-group expression data

Up to this point, we have restricted our analysis to datasets consisting of two groups of samples. While pairwise comparisons have been characterised as the bread and butter of differential expression analyses, multi-group datasets are also quite common. In this section, we demonstrate the applicability of *DGEclust* for such datasets by using the CAGE data from human brains as a test case. *DGEclust* processed the data for 10K iterations, of which the first 5K were rejected as burn-in, while the remaining 5K were used for estimating differential expression, as outlined in a previous section. We also applied *edgeR* and *DESeq2* on the same dataset. *baySeq* was excluded from this analysis, because this would require examining a large number of possible expression patterns, which we found impractical.

**Figure 7:**
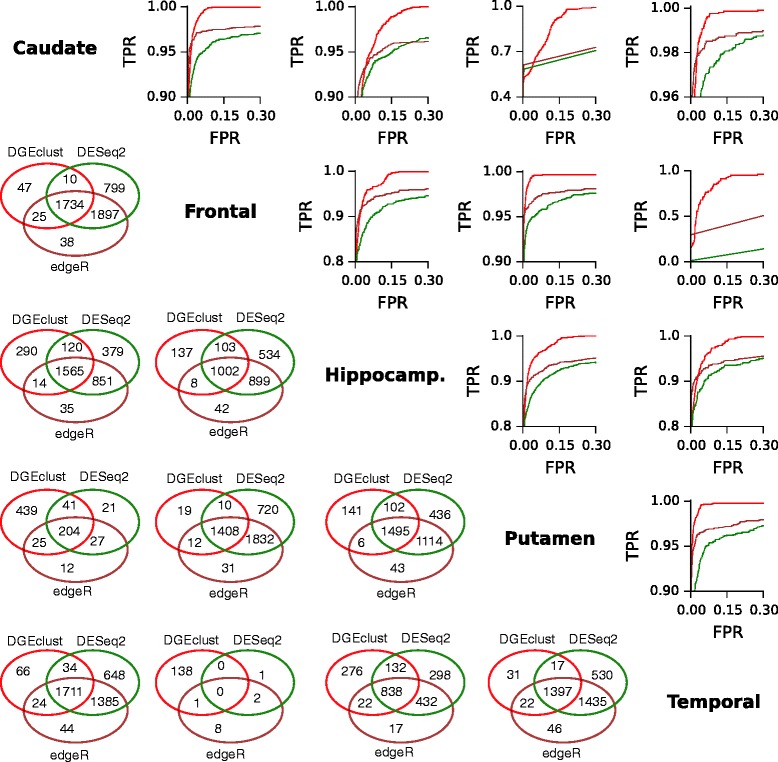
**Comparison of ROC curves for different methods.** The methods were applied to CAGE data from different regions of the human brain. A ground truth was established as in Figure 6. *DGEclust* is top-ranked in all cases. All methods demonstrate excellent performance, achieving a TPR larger than 0.8 at FPRs less than 0.01. As indicated by the Venn diagrams constructed from DE genes obtained at an FDR equal to 0.1*%*, the three methods demonstrate a significant overlap, sharing more than 1K genes in most cases. In terms of the number of novel discoveries (i.e. DE genes identified as such only by a particular method), *DGEclust* occupies the middle spot between *DESeq2* (first) and *edgeR* (third). CAGE, cap analysis of gene expression; DE, differentially expressed; FDR, false discovery rate; FPR, false positive rate; hippocamp., hippocampus; ROC, receiver operating characteristic; TPR, true positive rate.

Having established a ground truth as outlined in an earlier section, we constructed ROC curves for all possible pairs of brain regions in the CAGE dataset. It may be observed (Figure 7, upper triangle) that, for all pairs of brain regions (with the exception of the caudate/putamen and frontal/temporal pairs), all three methods demonstrated excellent performance, reaching TPRs larger than 80*%* at FPRs smaller than 1*%*. In all cases, *DGEclust* was the top performer as indicated by the AUC, followed by *edgeR* and then *DESeq2* in all cases. Examination of the Venn diagrams constructed from the DE genes, which were identified by each method at an FDR cutoff of 10*%*, indicates a significant overlap between all three methods, with at least 1K genes commonly identified as DE in most cases. In terms of novel discoveries (i.e. genes that were not called as DE by alternative methods), *DGEclust* appears to occupy the middle spot between *DESeq2* and *edgeR*, with *DESeq2* calling the largest number of novel discoveries in most cases. Two interesting exceptions were the caudate nucleus/putamen pair and the temporal/frontal lobes, for which *DGEclust* appears to have called the largest number of novel DE genes.

To validate these results further, we clustered the genes identified as DE by each method between at least one pair of brain regions at an FDR of 10*%* and we tested the validity of the resulting clusterings using the BHI [53] as a quality measure. BHI exploits gene ontology (GO) annotations to provide a measure of how biologically homogeneous a given clustering partition is. Clusters where many genes share annotations will lead to a high BHI score and vice versa. BHI values range from 0 to 1, with a score of 1 indicating the highly unlikely situation of perfect agreement for all GO terms. If the results in Figure 7 are valid, we expect the BHI score computed for *DGEclust* to be at least as high as the score computed for the other methods.

For clustering the DE genes called by each method, we used the *k*-means algorithm with $\sqrt {N_{\textit {DE}}/2}$ clusters as input, where *N*_*DE*_ is the number of DE genes. We could have used more elaborate methods for choosing the optimal number of clusters, such as Akaike’s information criterion or the Bayesian information criterion, but for our purposes, this simple heuristic suffices. We also used *hierarchical clustering* with an average linkage and a Euclidean distance metric. An optimal clustering partition was subsequently extracted by cutting the resulting hierarchical clustering (visualised as a dendrogram) at a distance of 0.5, which optimises the expectation of Binder’s loss function [54]. In both cases, the raw counts were log-transformed before clustering using *DESeq2*’s rlog function.

For *DGEclust*, we additionally performed hierarchical clustering using a similarity matrix computed internally by our software. Specifically, each element $s_{ii^{\prime }}$ of this matrix measuring the similarity between genes *i* and *i*^′^ is defined as follows:

(8) $$ s_{ii^{\prime}} = \frac{\sum_{t=T_{0}+1}^{T_{0}+T} \frac{\sum_{l=1}^{L} 1\!\!1\left(z_{il}^{(t)}=z_{i'l}^{(t)}\right)}{L}}{T}  $$

where *L* is the number of brain regions in the dataset.

Our results from this analysis are indicated in Table 1. As a general observation, the BHI scores are rather low, with only the overall BHI score reaching values of 0.20 to 0.21, while the BHI scores for each individual GO domain range between 0.05 and 0.09. As expected, *DGEclust* supports BHI scores at least as good as those computed for the other methods (indicated in bold in Table 1). Specifically, for the biological process and cellular component GO domains, *DGEclust* has the highest score of 0.07 and 0.09, respectively, while for molecular function and overall BHI, the scores (0.08 and 0.21, respectively) are no worse than those returned from the other methods. This indicates that the clusters supported by the DE genes called by *DGEclust* are at least as biologically homogeneous as those supported by the other methods, thus increasing our confidence in the ability of *DGEclust* to identify DE genes correctly as indicated in Figure 7.

**Table 1:** **Biological homogeneity index scores for the CAGE dataset**

**Software**	**Clustering**	**Number**	**Number**	**BHI (BP)**	**BHI (CC)**	**BHI (MF)**	**BHI (all)**
		**of DE genes**	**of clusters**				
*DGEclust*	Hierarchical	2,177	1	**0.07**	0.08	**0.08**	**0.21**
	Hierarchical ^∗^		17	0.05	**0.09**	0.07	0.20
	*k*-means		32	0.05	0.07	0.08	0.20
*DESeq2*	Hierarchical	7,109	1	0.06	0.08	0.08	0.20
	*k*-means		59	0.06	0.08	0.07	0.21
*edgeR*	Hierarchical	5,705	1	0.06	0.08	0.08	0.20
	*k*-means		53	0.06	0.07	0.08	0.21

We computed the BHI scores for each GO domain (biological process, molecular function and cellular component), as well as an overall score. *k*-means and hierarchical clustering were applied to the regularised log-transformed counts for all genes that were called DE between at least one pair of brain regions by each of the three examined methods, i.e. *DGEclust*, *DESeq2* and *edgeR*. For *k*-means, we used an optimal number of clusters equal to $\sqrt {N_{\textit {DE}}/2}$, where *N*_*DE*_ is the number of DE genes. For the hierarchical clustering, we used average linkage and a Euclidean distance metric with a cutoff distance of 0.5 to obtain an optimal clustering. For *DGEclust*, we also applied hierarchical clustering using an internally computed similarity matrix. This is indicated with an asterisk (^∗^). The highest score in each GO domain is indicated in bold. BHI, biological homogeneity index; BP, biological process; CC, cellular component; DE, differentially expressed; GO, gene ontology; MF, molecular function.

**Figure 8:**
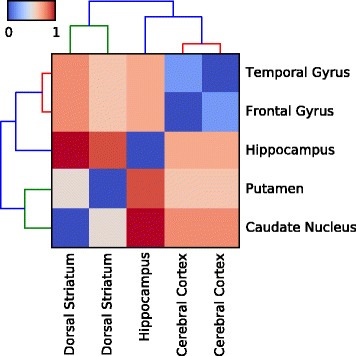
**Hierarchical clustering of brain regions based on CAGE data.** We constructed a similarity matrix based on the number of differentially expressed transcripts discovered by *DGEclust* between all possible pairs of brain regions. This similarity matrix was then used as input to a hierarchical clustering algorithm using a Euclidean distance metric and average linkage. As illustrated by the generated heat map and dendrograms, cortical regions (frontal and temporal lobes) are clustered together with the hippocampus and all three are maximally distant from subcortical regions, i.e. the dorsal striatum (putamen and caudate nucleus) of the basal ganglia. CAGE, cap analysis of gene expression.

Furthermore, we investigated the relation between different brain regions by constructing a similarity matrix, which we used as input to hierarchical clustering routines for the generation of dendrograms and heat maps. Every element $s_{ll^{\prime }}$ of this matrix measures the similarity between brain regions *l* and *l*^′^ and it is computed as follows:

(9) $$ s_{ll^{\prime}} = \frac{\sum_{i=1}^{N}\pi_{i}}{N}|_{ll^{\prime}}   $$

where *N* is the number of genes in the dataset. The similarity matrix calculated as above was used to construct the dendrograms and heat map in Figure 8, after employing a Euclidean distance metric and average linkage. It may be observed that the resulting hierarchical clustering reflects the evolutionary relations between different brain regions. For example, the temporal and frontal lobe samples, which are both located in the cerebral cortex, are clustered together. The hippocampus, albeit lying beneath the cerebral cortex and much older and more primitive than the surrounding neocortex, is not truly a sub-cortical structure, but rather a cortical infolding. Thus, it is clustered together with the temporal and frontal samples. The subcortical caudate nucleus and putamen, which form the dorsal striatum – an important part of the basal ganglia – are clustered together and they are maximally distant from the cortical structures.

Collectively, the evidence presented in Figure 8 and in Table 1 is supportive of the improved capacity of *DGEclust* for calling DE genes, as presented in Figure 7.

### *DGEclust* has reasonable computational requirements

Bayesian approaches employing MCMC sampling methodologies for inference are notorious for their increased computational requirements. Being conscious that this might discourage application of this class of methodologies to large genomic datasets, we investigated how our method scales with increasing number of samples and genes. Specifically, we used *IPython*’s %memit and %timeit commands to measure peak memory usage and computation time. All simulations were performed on a MacBook Pro with an Intel four-core i7 processor and 8 Gb of memory.

**Figure 9:**
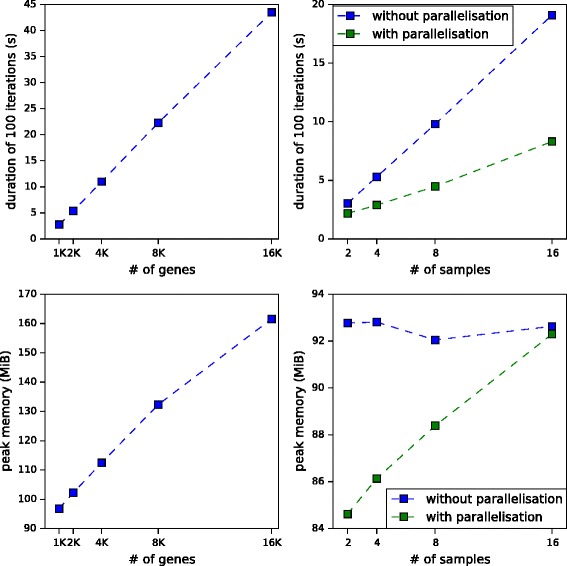
**Computational requirements of** ***DGEclust*** **.** Computation time and peak memory usage scale linearly with the number of genes and with the number of clusters. These measurements were obtained using *IPython*’s %timeit and %memit commands. Using multiple cores to process samples has a significant impact on simulation speeds (top right panel). Genes are processed in parallel by default.

As indicated in Figure 9, computation time and peak memory usage increase linearly with the number of genes (left top and bottom panels). Operations on genes are performed in parallel by default extensively using *NumPy*’s vector notation and, for this reason, top performance in terms of speed is expected. Groups of samples can also be processed in parallel using multiple cores, if the user wishes to do so. Peak memory usage and computation time also increase linearly with the number of samples (right top and bottom panels), although peak memory usage appears roughly constant over the examined range for the number of samples in the absence of multiprocessing. There is a clear gain in processing speed from using multiple cores to process samples (top right panel). In conclusion, these data suggest that *DGEclust* is fundamentally applicable even for large genomic datasets, particularly when multiple cores are used.

## Conclusions

Despite the availability of several protocols (e.g. single vs paired-end) and sequencing equipment (e.g. Solexa’s Illumina Genome Analyzer, ABI Solid Sequencing by Life Technologies and Roche’s 454 Sequencing), all NGS technologies follow a common set of experimental steps (see [7] for a review) and, eventually, generate data, which essentially constitute a discrete, or *digital* measure of gene expression. These data are fundamentally different in nature (and, in general terms, superior in quality) from the continuous fluorescence intensity measurements obtained from the application of microarray technologies. In comparison, NGS methods offer several advantages, including detection of a wider level of expression levels and independence of prior knowledge of the biological system, which is required by hybridisation-based microarrays [7]. Due to their better quality, NGS assays have tended to replace microarrays, despite their higher cost [55].

In this article, we have addressed the important issues of clustering and differential expression analysis of digital expression data and we demonstrate the intimate relation between these two concepts. Most proposals for clustering RNA-seq and similar types of data have focused on clustering variables (i.e. biological samples), instead of features (e.g. genes) and they employ distance-based or hierarchical clustering methodologies on appropriately transformed datasets, e.g. [24,56,57]. For example, the authors in [24] calculate a common variance function for all samples in a tag-seq dataset of glioblastoma-derived and non-cancerous neural stem cells using a variance-stabilising transformation, followed by hierarchical clustering using a Euclidean distance matrix. In [56], a Pearson correlation dissimilarity metric was used for the hierarchical clustering of RNA-seq profiles for 14 different tissues from soybean after these were normalised using a variation of the RPKM method [5,6].

The above approaches, although fast and relatively easy to implement, do not always take into account the discrete nature of digital gene expression data. For this reason, various authors have developed distance metrics based on different parameterisations of the log-linear Poisson model for modelling count data, e.g. [58-60]. A more recent class of methods follows a model-based approach, where the digital dataset is modelled as a random sample from a finite mixture of discrete probability distributions, usually Poisson or negative binomial [61-63]. Using a full statistical framework for describing the observed count data, these model-based approaches often perform better than distance-based algorithms, such as *k*-means [61].

Although computationally efficient and attractive due to their relative conceptual simplicity, the utility of both distance- and finite model-based clustering methods has been criticised [19,41]. One particular feature of these methodologies, which compromises their applicability, is that the number of clusters in the data must be known *a priori*. For example, both the *k*-means and the self-organising map algorithms require the number of clusters as input. Similarly, methods that model the data as a finite mixture of Poisson or negative binomial distributions [61-63] require prior knowledge of the number of mixture components. Estimating the number of clusters usually makes use of an optimality criterion, such as the Bayesian information criterion or the Akaike information criterion, which requires repeated application of the algorithm on the same dataset with different initial choices of the number of clusters. Thus, the number of clusters and the parameters for each individual cluster are estimated separately, making the algorithm sensitive to the initial model choice. Similarly, hierarchical clustering methods often rely on some arbitrary distance metric (e.g. Euclidean or Pearson correlation) to distinguish between members of different clusters, without providing a criterion for choosing the correct number of clusters or a measure of the uncertainty of a particular clustering, which would serve to assess its quality.

In this article, we have developed a statistical methodology and associated software (*DGEclust*) for clustering digital gene expression data, which (unlike previously published approaches [56-60]) does not require any prior knowledge of the number of clusters, rather it estimates this parameter and its uncertainty simultaneously with the parameters (e.g. location and shape) of each individual cluster. This is achieved by embedding the negative binomial distribution for modelling count data in a hierarchical Dirichlet process mixture framework. Our formulation implies that distributional parameters (i.e. fold-changes) are not all distinct, but they are shared between genes and between groups of samples. This is a form of information sharing between genes and between samples, which is made possible by the particular hierarchical structure of the proposed model. At each level of the hierarchy, the number of mixture components, i.e. the number of clusters, is assumed infinite. This represents a substantial departure from previously proposed finite mixture models and avoids the need for arbitrary prior choices regarding the number of clusters in the data.

Despite the infinite dimension of the mixture model, only the finite number of clusters supported by the data and the associated parameters are estimated. This is achieved by introducing a blocked Gibbs sampler, which permits efficient processing of large datasets containing more than 20K genes. Unlike MCMC inference methods for HDPMM based on the popular Chinese restaurant franchise metaphor [16,41], our algorithm allows all gene-specific parameters in each sample to be updated simultaneously and independently from other samples. This allows rapid convergence of the algorithm and permits the development of parallelised implementations of the Gibbs sampler, which enjoy the increased performance offered by modern multicore processors.

The second important contribution of this study is demonstrating how this type of hierarchical clustering can be used for differential expression analysis. We emphasise that differential expression can be thought of as a particular form of clustering. Through comparison with popular alternatives for both simulated and actual experimental data, we demonstrate the applicability of this approach for a wide range of experimental settings and its improved performance, particularly at small sample sizes, which reflects the design of current sequencing experiments.

In conclusion, we have developed a hierarchical, non-parametric Bayesian method for modelling digital expression data. The novelty of our method is simultaneously addressing the problems of model selection and estimation uncertainty and exposing the intimate relation between clustering and differential expression analysis. We expect our work to inspire and support further theoretical research on modelling digital expression data and we believe that our software, *DGEclust*, will prove to be a useful addition to the existing tools for the statistical analysis of RNA-seq and similar types of data.

## Materials and methods

We implemented the methodology presented in this article in the software package *DGEclust*, which is written in Python and uses the SciPy stack. *DGEclust* expects as input and clusters a matrix of unnormalised count data along with replication information, if this is available. The output of the clustering process is required as input to post-processing routines, which compute the posterior probabilities that each particular gene between any two groups of samples in the data are DE. Further post-processing routines can generate gene- or library-wise similarity matrices, which can be used as input to hierarchical clustering routines for the generation of heat maps and dendrograms. *DGEclust* takes advantage of multicore processors to accelerate computations. All analyses in this article were performed using *DGEclust* and standard Python/SciPy tools, as well as *DESeq*/*DESeq2*, *edgeR* and *baySeq* for comparison purposes. When using these packages, all parameters were left at their default values. For *baySeq*, we used 5K samples when estimating the priors using the quasi-likelihood approach. To make a comparison possible, we processed all datasets with *DGEclust* for 10K iterations, rejecting the first 5K of them as burn-in.

### Normalisation

Internally, *DGEclust* uses the same normalisation method as *DESeq*. This behaviour can be overridden by providing a set of library sizes as input. When comparing different software packages, we used the default normalisation method of each package.

### CAGE library preparation and data pre-processing

Post-mortem human brain tissue from frontal, temporal, hippocampus, caudate and putamen regions from five donors was obtained from the Netherlands Brain Bank (NBB, Amsterdam, Netherlands). All tissue requests received at the NBB are reviewed by the NBB’s scientific committee and all materials and data collected are obtained with written informed consent. The procedures, information and consent forms of the NBB have been approved by the Medical Ethics Committee of the VU Medical Centre (Amsterdam, Netherlands).

Total RNA was extracted and purified using the Trizol tissue kit according to the manufacturer’s instructions (Invitrogen, Waltham, Massachusetts, USA). CAGE libraries were prepared according to the standard Illumina CAGE protocol [51]. Briefly, 5 µg of total RNA was reverse transcribed with reverse transcriptase. Samples were cap-trapped and a specific linker, containing a 3-bp recognition site and the type III restriction-modification enzyme EcoP15I, was ligated to the single-strand cDNA. The priming of the second strand was done with specific primers. After synthesis of the second strand and cleavage with EcoP15I, another linker was ligated. Purified cDNA was then amplified with 10 to 12 PCR cycles. PCR products were purified, their concentration was adjusted to 10 nM and they were sequenced on a HiSeq 2000 using the standard protocol for 50-bp single-end runs.

Sequenced reads (tags) were filtered for known CAGE artefacts using TagDust [64]. Low quality reads and reads mapping to known rRNA were also removed. The remaining reads were mapped to the human genome (build hg19) using the Burrows–Wheeler aligner for short reads [65]. Mapped reads overlapping or located within 20 bp on the same strand were grouped into tag clusters and tag clusters with low read counts were removed.

### URL

The most recent version of *DGEclust* is available online under the MIT licence [66].

## Additional file

Additional file 1 **Supplementary Material for DGEclust: differential expression analysis of clustered count data.** In this supplementary material we provide a detailed account of posterior inference in the model summarised by Equations 5.

## Abbreviations

AUC: area under the receiver operating characteristic curve
BHI: biological homogeneity index
bp: base pair
CAGE: cap analysis of gene expression
DE: differentially expressed
FD: false discovery
FDR: false discovery rate
FPR: false positive rate
GO: gene ontology
HDP: hierarchical Dirichlet process
HDPMM: hierarchical Dirichlet process mixture model
MCMC: Markov chain Monte Carlo
NBB: Netherlands Brain Bank
NGS: next-generation sequencing
RNA-seq: RNA sequencing
ROC: receiver operating characteristic
SAGE: serial analysis of gene expression
TPR: true positive rate
