# Supplementary material for: DGEclust: differential expression analysis of clustered count data
Source: Genome Biol. 2015 Feb 20;16(1):39. doi: 10.1186/s13059-015-0604-6 (PMC4365804; doi:10.1186/s13059-015-0604-6)
Supplement: Supplementary file 1 — Supplementary Material for DGEclust: differential expression analysis of clustered count data. In this supplementary material we provide a detailed account of posterior inference in the model summarised by Equations 5. [file 13059_2015_604_MOESM1_ESM.pdf]

Supplementary Material for "DGEclust: differential expression analysis of clustered count data"

Dimitrios V Vavoulis*[1] and Margherita Francescatto[2] and Peter Heutink[2] and Julian Gough*[1]

[1]Department of Computer Science, University of Bristol, Bristol, UK
[2]Genome Biology of Neurodegenerative Diseases, Deutsches Zentrum für Neurodegenerative Erkrankungen, Tübingen, Germany.

Email: Dimitris.Vavoulis@bristol.ac.uk; Margherita.Francescatto@dzne.de; Peter.Heutink@dzne.de; Julian.Gough@bristol.ac.uk;

*Corresponding author

Introduction

In this Supplementary Material, we provide the mathematical details of the algorithm we used to make posterior inferences on the generative model described by Eqs. 5. Currently, there are two major classes of methods for posterior inference in Hierarchical Dirichlet Process Mixture Models. The first class includes algorithms based on the Chinese Restaurant metaphor for representing the Dirichlet Process, as for example in [1, 2]. The second class includes variational inference approaches in either a batch or an online data processing context, as for example in [3]. The algorithm we present here is an extension of the blocked Gibbs sampler in [4] (which was developed for non-–hierarchical Dirichlet Process mixture models) in the case of non–conjugate Hierarchical Dirichlet Process mixtures. Unlike variational methodologies, it approximates exact posteriors and, unlike approaches based on the Chinese Restaurant metaphor, it follows a blocked sampling strategy, which permits, in principle, faster convergence, and parallel implementation on multicore processors, which opens the possibility for applications on large gene expression datasets.

The first step in developing this algorithm is to cast the implicit generative model summarised in Eqs. 5 in an equivalent, explicit form, which makes use of Sethuraman's constructive representation of a draw from

the Dirichlet Process [3, 5]:

$$
\begin{aligned}
\beta'_k &\sim \text{Normal}\left(\mu_\beta, \sigma^2_\beta\right) \\
w_k &\sim \text{Stick}(1, \delta), \qquad k = 1, 2, \ldots \\
u_{rl} &\sim \text{Stick}(1, \gamma_l), \qquad r = 1, 2, \ldots \\
c_{rl} &\sim \text{Categorical}(w) \\
d_{il} &\sim \text{Categorical}(u_l) \\
z_{il} &\equiv c_{d_{il}l} \\
\beta_{il} &\equiv \beta'_{z_{il}} \\
\log(\phi_i) &\sim \text{Normal}\left(\mu_\phi, \sigma^2_\phi\right) \\
y_{ij}|\phi_i, \mu_i, \beta_{il} &\sim \frac{\Gamma(y_{ij} + \alpha_i)}{\Gamma(\alpha_i)\Gamma(y_{ij} + 1)} \left(\frac{\alpha_i}{\alpha_i + m_{ij}}\right)^{\alpha_i} \left(\frac{m_{ij}}{\alpha_i + m_{ij}}\right)^{y_{ij}}
\end{aligned} \tag{1}
$$

where $l \equiv \lambda(j)$, $\alpha_i \equiv \phi_i^{-1}$ and $\log(m_{ij}) = \log(c_j) + \log(\mu_i) + \beta_{il}$. This representation introduces matrices of group– and gene–specific indicator variables, $c = \{c_{rl}\}$ and $d = \{d_{il}\}$, with elements sampled from the categorical distribution with weights $w = \{w_k\}$ (a vector) and $u = \{u_{rl}\}$ (a matrix), respectively. What makes the above model special is how we generate the weights w and u, which resembles a stick–breaking process; for w, we set $w_1 = V_1$ and $w_k = V_k \prod_{k'=1}^{k-1}(1 - V_{k'})$ for $k \geq 2$, where $V_1, \ldots, V_k$ are random variables following a Beta distribution, $V_k \sim \text{Beta}(1, \delta)$. For the sake of brevity, we write $w_k \sim \text{Stick}(1, \delta)$. Similarly, $u_{rl} \sim \text{Stick}(1, \gamma_l)$, for Beta–distributed variables sampled as $V_{rl} \sim \text{Beta}(1, \gamma_l)$.

We wish to compute the joint posterior of the various random variables that appear in the model, $\text{p}(\phi, \mu, \beta', c, d, w, u, \delta, \gamma, \mu_\beta, \sigma^2_\beta, \mu_\phi, \sigma^2_\phi|y)$, where y is a matrix of count data; ϕ and μ are vectors of gene–specific dispersion and mean expression parameters, respectively; β' is a vector of log–fold changes; δ and γ are concentration parameters (a scalar and a vector, respectively); and μ_β, μ_ϕ, σ^2_β and σ^2_ϕ are scalar hyper–parameters. We approximate the above distribution through numerical (Monte Carlo) methods, i.e. by sampling a large number of $\{\phi, \mu, \beta', c, d, w, u, \delta, \gamma, \mu_\beta, \sigma^2_\beta, \mu_\phi, \sigma^2_\phi\}$–tuples from it. One way to achieve this is by constructing a Markov chain, which admits $\text{p}(\phi, \mu, \beta', c, d, w, u, \delta, \gamma, \mu_\beta, \sigma^2_\beta, \mu_\phi, \sigma^2_\phi|y)$, as its stationary distribution. Such a Markov chain can be constructed by using Gibbs sampling, which consists of sampling repeatedly from the conditional posteriors for each of these random variables, given the most recent values of all other variables that appear in each conditional. Below, we explain how to sample from each of these conditionals. Notice that, with a slight abuse of notation, we use the symbol p to indicate either a probability density or a probability mass function, depending on the context. Similarly, conditionals are often indicated

as $\mathrm{p}(x|y,\cdot)$, where the variables which x is conditioned on (other than the data) are summarised by a dot $(\cdot)$ for simplicity. Finally, for a matrix x with elements x_{ij}, we indicate the i-th row and the j-th column as $x_{i\cdot}$ and $x_{\cdot j}$, respectively.

Updates for the vector of dispersion parameters, ϕ

Each element ϕ_i of the vector ϕ can be sampled independently from the others. If we let $y_{i\cdot}$ and $\beta_{i\cdot}$ be the vector of count data for gene i across all samples and the vector of group–specific log–fold changes for gene i, respectively, we can write:

$$\begin{aligned}\mathrm{p}(\phi_i|y_{i\cdot},\mu_i,\beta_{i\cdot}) &\propto \mathrm{p}(y_{i\cdot}|\phi_i,\mu_i,\beta_{i\cdot})\mathrm{p}(\phi_i|\mu_\phi,\sigma^2_\phi) \\ &\propto \prod_{j=1}^{M}\mathrm{p}(y_{ij}|\phi_i,\mu_i,\beta_{i\lambda(j)})\mathrm{p}(\phi_i|\mu_\phi,\sigma^2_\phi) \end{aligned} \tag{2}$$

where the likelihood and the prior are a negative binomial and a log–normal distribution, respectively, as indicated in Eqs. 1. Since the posterior of ϕ_i cannot be sampled from directly, we employ a Metropolis step with proposals ϕ_i^* sampled from the prior of ϕ_i and acceptance probability equal to:

$$\mathrm{p}_{acc} = \min\left(1, \frac{\prod_{j=1}^{M}\mathrm{p}(y_{ij}|\phi_i^*,\mu_i,\beta_{i\lambda(j)})}{\prod_{j=1}^{M}\mathrm{p}(y_{ij}|\phi_i,\mu_i,\beta_{i\lambda(j)})}\right) \tag{3}$$

Notice that, since we use the prior for sampling new proposals, the acceptance probability merely involves the ratio of two likelihoods.

Updates for the vector of mean expression levels, μ

As in the case of ϕ_i, the gene–specific expression levels μ_i can be sampled independently to each other. We observe that instead of working directly with μ_i, it is easier to work with the proxy probability variable $p_i = \alpha_i/(\alpha_i+\mu_i)$, where $\alpha_i \equiv \phi_i^{-1}$. Assuming a non–informative Beta prior for p_i, $\mathrm{p}(p_i) = \mathrm{Beta}(0.5, 0.5)$, we can write:

$$\begin{aligned}\mathrm{p}(p_i|\tilde{y}_{i\cdot},\phi_i) &\propto \mathrm{p}(\tilde{y}_{i\cdot}|\phi_i,p_i)\mathrm{p}(p_i) \\ &\propto \prod_{j=1}^{M}\mathrm{p}(\tilde{y}_{ij}|\phi_i,p_i)\mathrm{p}(p_i)\end{aligned} \tag{4}$$

where $\tilde{y}_{ij} = y_{ij}/(c_j\beta_{i\lambda(j)})$ and $\mathrm{p}(\tilde{y}_{ij}|p_i,\phi_i) \propto p_i^{\alpha_i}(1-p_i)^{\tilde{y}_{ij}}$. It follows that the posterior of p_i is a Beta distribution:

$$\mathrm{p}(p_i|\tilde{y}_{i\cdot},\phi_i) = \mathrm{Beta}(0.5+c_1, 0.5+c_2) \tag{5}$$

where $c_1 = \sum_{j=1}^{M} \tilde{y}_{ij}$, $c_2 = M/\phi_i$ and M is the number of libraries. A sample from the posterior of μ_i is then obtained by a simple transformation of the newly sampled p_i:

$$\mu_i = \frac{1 - p_i}{\phi_i p_i} \tag{6}$$

Updates for the vector of weights, w

In Eq. 1, the indices k for the sequence of weights $w = \{w_k\}$ go from 1 to ∞. In practice, we truncate this infinite series by rejecting all terms with index larger than K and setting $w_K = 1 - \sum_{k=1}^{K-1} w_k$, which is equivalent to setting $V_K = 1$. It is known that for sufficiently large values of K, the above truncation is virtually indistinguishable from the full (infinite) sequence [4].

The first step in updating the truncated sequence of weights w is generating $K - 1$ Beta–distributed random numbers, as follows:

$$V_k \sim \text{Beta}\left(1 + N_k, \delta + NL - \sum_{k'=1}^{k} N_{k'}\right), \qquad k = 1, \ldots, K - 1 \tag{7}$$

where N is the number of genes, L is the number of library groups and N_k is the number of elements of z that equal k. Notice that z is a matrix with elements $z_{il} = c_{d_{il}l}$. Also, $V_K = 1$ in order to ensure that the weights add up to 1. These are then simply generated by setting $w_1 = V_1$ and $w_k = V_k \prod_{k'=1}^{k-1}(1 - V_{k'})$ for $k \geq 2$, as mentioned in a previous section.

Updates for the matrix of weights, u

Similarly to the case of w, the matrix of gene– and group–specific weights $u = \{u_{rl}\}$ is truncated by rejecting all elements for which r is larger than R. For each group of libraries l, the vector of weights $u_{\cdot l}$ can be updated as described above for w. We begin by sampling $K - 1$ Beta–distributed random numbers:

$$V_{rl} \sim \text{Beta}\left(1 + N_{rl}, \gamma_l + N - \sum_{r'=1}^{r} N_{r'l}\right), \qquad r = 1, \ldots, R - 1 \tag{8}$$

where N_{rl} is the number of elements of the vector of indicators $d_{\cdot l}$ equal to r, N is the number of genes and $V_R = 1$. The weights $u_{\cdot l}$ are then generated by a stick–breaking process, as described above for w.

Updates for the matrix of indicator variables, d

Each gene– and group–specific element d_{il} in the matrix of indicator variables d takes values between 1 and R and it can be updated independently to the others. The posterior probability that d_{il} is equal to r for

$r = 1, \ldots, R$ is:

$$\mathrm{p}(d_{il} = r | y^l_{i\cdot}, \phi_i, \mu_i, \beta', c_{\cdot l}, u_{\cdot l}) \propto \prod_{j=1}^{M_l} \mathrm{p}(y^l_{ij} | \phi_i, \mu_i, \beta'_{c_{rl}}) u_{rl} \tag{9}$$

where $y^l_{i\cdot}$ is the set of expression measurements for gene i in the group of libraries l, u_{rl} is the prior probability that $d_{il} = r$ and M_l is the number of libraries in group l. A straightforward way to sample from the posterior of d_{il} is to compute the posterior probabilities $\pi_{ilr} = \mathrm{p}(d_{il} = r | y^l_{i\cdot}, \cdot)$ for all possible values of r and then sample d_{il} from a Categorical distribution with weights $\tilde{\pi}_{il1}, \ldots, \tilde{\pi}_{ilR}$, where $\tilde{\pi}_{ilr} = \pi_{ilr} / \sum_{r'=1}^{R} \pi_{ilr'}$.

Updates for the matrix of indicator variables, c

The elements c_{rl} of matrix c take values from 1 to K and they are sampled independently to each other, similarly to u. We start by writing the posterior probability that c_{rl} is equal to k:

$$\mathrm{p}(c_{rl} = k | y^l, \phi, \mu, \beta', d_{\cdot l}, w) \propto \prod_{i=1}^{N} \prod_{j=1}^{M_l} \mathrm{p}(y^l_{ij} | \phi_i, \mu_i, \beta'_k)^{[d_{il} = r]} w_k \tag{10}$$

where y^l is the $N \times M_l$ matrix of counts corresponding to group of libraries l, w_k is the prior probability that $c_{rl} = k$ and $[\cdot]$ is equal to 1 if the expression inside the square brackets is true and 0 otherwise. A sample from the posterior of c_{rl} is obtained by computing the posterior probabilities for all possible values of k, normalising and sampling from a Categorical distribution, similarly to the case of d_{il} above.

Updates for the vector of log–fold changes, β'

The posterior density of each element β'_k of β' is as follows:

$$\mathrm{p}(\beta'_k | y, \phi, \mu, c, d) \propto \prod_{i=1}^{N} \prod_{j=1}^{M} \mathrm{p}(y_{ij} | \phi_i, \mu_i, \beta'_k)^{[z_{i\lambda(j)} = k]} \mathrm{p}(\beta'_k | \mu_\beta, \sigma^2_\beta) \tag{11}$$

where the prior $\mathrm{p}(\beta'_k | \cdot)$ is normal and $z_{il} = c_{d_{il} l}$ as indicated in Eqs. 1. In order to sample from the above posterior, we employ a Metropolis step with proposals β'^{*}_k sampled from the prior and acceptance probability:

$$\mathrm{p}_{acc} = \min \left(1, \frac{\prod_{i=1}^{N} \prod_{j=1}^{M} \mathrm{p}(y_{ij} | \phi_i, \mu_i, \beta'^{*}_k)^{[z_{i\lambda(j)} = k]}}{\prod_{i=1}^{N} \prod_{j=1}^{M} \mathrm{p}(y_{ij} | \phi_i, \mu_i, \beta'_k)^{[z_{i\lambda(j)} = k]}} \right) \tag{12}$$

Notice that for those k for which the condition $z_{i\lambda(j)} = k$ is never true, the corresponding β'_k is simply sampled from the prior of β'.

Updates for the concentration parameters δ and γ

The concentration parameter δ and the vector of concentration parameters γ influence the expected number of clusters of the corresponding Dirichlet processes. Sampling from the posterior of the concentration parameter

of the Dirichlet process has been covered previously in [6] and in [4], which is the approach we follow here.

Assuming a Gamma prior with shape a and rate b, the posterior of δ depends only on the vector of weights w:

$$\mathrm{p}(\delta|w) = \mathrm{Gamma}(a + K - 1, b - \log(w_K)) \tag{13}$$

Similarly, for each element γ_l of γ, the posterior becomes:

$$\mathrm{p}(\gamma_l|u_l) = \mathrm{Gamma}(a + R - 1, b - \log(u_{Rl})) \tag{14}$$

Both δ and the elements of γ are sampled independently to each other.

Updates for the hyper–parameters μ_β, σ^2_β, μ_ϕ and σ^2_ϕ

Assuming non–informative priors, the marginal posteriors of μ_ϕ and σ^2_ϕ are normal and inverse gamma, respectively [7]:

$$\begin{aligned}\mathrm{p}(\mu_\phi|\phi, \sigma^2_\phi) &= \mathrm{Normal}\left(\bar{m}_\phi, \frac{\sigma^2_\phi}{N}\right)\\ \mathrm{p}(\sigma^2_\phi|\phi) &= \mathrm{InvGamma}\left(\frac{N}{2}, \frac{s^2_\phi}{2}\right)\end{aligned} \tag{15}$$

where $\bar{m}_\phi = \sum_{i=1}^N \log(\phi_i)/N$, $s^2_\phi = \sum_{i=1}^N (\log(\phi_i) - \bar{m}_\phi)^2$ and N is the number of genes.

Similarly, the marginal posteriors for μ_β and σ^2_β are:

$$\begin{aligned}\mathrm{p}(\mu_\beta|\beta', \sigma^2_\beta) &= \mathrm{Normal}\left(\bar{m}_\beta, \frac{\sigma^2_\phi}{K'}\right)\\ \mathrm{p}(\sigma^2_\beta|\beta') &= \mathrm{InvGamma}\left(\frac{K'}{2}, \frac{s^2_\beta}{2}\right)\end{aligned} \tag{16}$$

where $\bar{m}_\beta = \sum_{k=1}^{K'} \beta'_k/K'$ and $s^2_\beta = \sum_{k=1}^{K'} (\beta'_k - \bar{m}_\beta)^2$. Notice that only the β'_k for which the condition $z_{i\lambda(j)} = k$ is true for at least one pair of i and j participate in the above computation. K' is the number of these β'_k.

Summary

Having determined the methodology for sampling from the various conditional posterior distributions, we can now summarise the algorithm for sampling from the full joint posterior $\mathrm{p}(\phi, \mu, \beta', c, d, w, u, \delta, \gamma, \mu_\beta, \sigma^2_\beta, \mu_\phi, \sigma^2_\phi|y)$. Starting with a set of initial values for all random variables in the model, we repeat the following steps:

1. Sample ϕ_i for all i independently given the most recent values of all other variables
2. Sample μ_i for all i independently given the most recent values of all other variables
3. Sample β'_k for all k independently given the most recent values of all other variables
4. Sample c_{rl} for all r and l independently given the most recent values of all other variables
5. Sample d_{il} for all i and l independently given the most recent values of all other variables
6. Sample δ and γ_l for all l independently given the most recent values of all other variables
7. Sample σ^2_ϕ and σ^2_β independently given the most recent values of all other variables
8. Sample μ_ϕ and μ_β independently given the most recent values of all other variables

The initial values of the various variables that appear in the model do not seem to affect the final equilibrium state, but they might influence the speed of convergence. Truncation levels were $K = R = 100$.

References

1. Teh YW, Jordan MI, Beal MJ, Blei DM: **Hierarchical Dirichlet processes**. *Journal of the American Statistical Association* 2006, **101**:1566–1581.
2. Wang L, Wang X: **Hierarchical Dirichlet process model for gene expression clustering**. *EURASIP J Bioinform Syst Biol* 2013, **2013**:5.
3. Wang C, Paisley J, Blei DM: **Online Variational Inference for the Hierarchical Dirichlet Process**. In *Proceedings of the 14th International Conference on Artificial Intelligence and Statistics* 2011.
4. Ishwaran H, James LF: **Gibbs Sampling Methods for Stick-Breaking Priors**. *Journal of the American Statistical Association* 2001, **96**(453):161–173, [http://www.jstor.org/stable/2670356].
5. SETHURAMAN J: **A CONSTRUCTIVE DEFINITION OF DIRICHLET PRIORS**. *Statistica Sinica* 1994, **4**:639–650.
6. West M: **Hyperparameter estimation in Dirichlet process mixture models**. Tech. Rep. 92-A03, Duke University 1992.
7. Yang R, Berger J: **A Catalogue of Noninformative Priors**. Tech. rep., Duke University 1997.
